# The Darenth Scandal

**Published:** 1898-02-05

**Authors:** 


### The Hospital

A Journal of

The Medical Sciences and Hospital Administration.

Vol. XXIII.—No. 593.] Edited by Sir Henry Burdett, K.C.B., and Solomon C. Smith, M.D., M.R.C.P. [Feb. 5, 1898.

### THE DARENTH SCANDAL.

The Metropolitan Asylums Board, at their meeting on Saturday last, had under consideration the circumstances of what has been called the "Darenth scandal," and came to the not very surprising conclusion that their proper course would be to make a scapegoat of the acting medical superintendent, Dr. White, who seems undoubtedly to have been remiss in his observance of forms, and thus to have furnished them with an excuse, if not with a reason, for the course which they have thought fit to adopt. The Darenth Asylum is a place for the reception of adult imbeciles of both sexes, and usually contains about a thousand. So long ago as last July one of the female inmates was discovered to be pregnant, but no official notice appears to have been taken of the fact, which was presumably well known to all who were likely to be concerned with it, and which was at once mentioned by Dr. White to the Chairman of the Committee. It would seem from the newspaper reports of the proceedings of the Asylums Board that the circumstance was not thought to be a matter of much importance, and that no special provision was made for the delivery, which occurred, with fatal issue, in November. The Committee of the Asylum reported to the Board that in their opinion Dr. White, who had had no obstetrical experience since his student days, prior to 1882, was unwise in undertaking what was likely to prove a difficult case; and they also found that there was no entry of the pregnancy in the case book until after the death, that the matter was not mentioned in the Medical Superintendent's written reports to the Committee, that no experienced nurse was provided, and that no communication was made to the coroner. The assigned cause of death was exhaustion after childbirth, and the report admits that Dr. White and his assistants appear to have spared no pains, and neglected no attention suggested by their experience, for the welfare of the patient during her confinement. Whether there were any conditions connected with the case, such for example as deformity of the pelvis, which were calculated to lead to any anticipation of difficulty, does not appear; but, even if there were, Dr. White's assistant might have been expected to be capable of dealing with them. Dr. White himself, who has been for some years in the asylum as assistant medical officer, passed his examinations before a knowledge of midwifery was rendered essential by the Act of 1886; and the Committee are probably right in their statement that his official life had given him no opportunity of gaining experience in this department of medicine. But his junior officer, who could only have recently been appointed, must have been in a different position; and the suggestion half made by the Committee, to the effect that the woman may have lost her life for want of sufficiently skilful attendance, seems on the face of it to be unwarrantable.

It does not appear from the newspaper reports at what date the poor woman entered the asylum, or what opportunities of leaving it may have been afforded her, and a motion for an inquiry into the early details of the affair, made by Mr. J. H. Brass, was ruled by the Chairman to be out of order. The high probability seems to be that the majority of the Darenth Committee, as well as the Chairman, knew all about the case through verbal communication; and that they were quite as blameworthy, in not reporting it officially to the Asylums Board, as Dr. White was in not reporting it officially to them. Dr. White was unquestionably wrong, and his books must have been imperfectly kept; but an error of this kind, although very likely to be productive of inconvenience in a public institution, does not seem to us to be of a very serious character, or to call for so severe a penalty as compulsory resignation. It may be assumed that, if the patient had not died, nothing would ever have been heard of the affair. It is easy to understand that a medical officer, whose heart is in the essentials of his work, may often be tempted to neglect book-keeping, or to postpone it to a more convenient season; and the Darenth case may be a useful lesson as to the danger of yielding to such temptation. All medical officers of public institutions should remember that they are under the authority of Boards or Committees mainly composed of persons who are capable of appreciating neatly-kept records, but who are not capable of appreciating the quality of professional work, and with whom, therefore, no excellence in the latter will outweigh negligence as to the former. Moreover, if Dr. White had reported the case in proper written form to his Committee, he would have removed all responsibility from his own shoulders to theirs. It would have been for them to consider the sufficiency or otherwise of the Asylum arrangements for a lying-in, and to afford any extraneous aid, in the way either of doctor or nurse, that they might have decided upon as necessary. In the meanwhile, we do not envy the feelings of the Chairman, who was informed of the pregnancy from the beginning, and who at least tacitly sanctioned the course pursued by Dr. White, which has led to the loss of a position which that gentleman had earned by sixteen years of work under the Asylums Board.

